# "The Hospital" Medical Book Supplement.—No. XV

**Published:** 1909-02-20

**Authors:** 


### "THE HOSPITAL" MEDICAL BOOK SUPPLEMENT.—No. XV.

CONTENTS.

**Syphilology—**
A System of Syphilis ... 543
Syphilis in the Army ... 543
**The Diseases of Children—**
Reports of the Society for the Study of Disease in Children ... 544
Some Characteristics and Requirements of Childhood ... 544
**Anæsthetics—**
Practical Points in Anæsthesia ... 544
**Otology—**
Infected Ears ... 544
**Ambulance Work—**
Aids to Memory for First-aid Students ... 545
Supplementary First-aid to Miners ... 545
**Advanced Physiology—**
Intracellular Enzymes ... 545
**Dental Surgery—**
Our Teeth ... 545
**Miscellaneous Items—**
Human Speech ... 546
The History of the Wine Trade in England ... 546
Glanders ... 546

### SYPHILOLOGY.

A SYSTEM OF SYPHILIS. In six volumes. Vol. 2. Edited by D'ARCY POWER and J. KEOGH MURPHY. (London : Henry Frowde and Hodder and Stoughton. 1908. £2 2s. net).

WHEN a man has paid twelve guineas for a treatise on a single disease, even so versatile and ubiquitous a malady as syphilis, he may reasonably expect a compendium of all that has been known of the subject up to the date of publication. He will not complain if he finds no final pronouncement upon the merits of the very latest fashion in treatment, but he may reasonably do so if there be not a pretty complete bibliography. From the favourable review of the first volume in these columns we withheld a suspicion that the "System" might not prove of the encyclopædic character to be hoped from the size and price of the volumes. The second volume, which we have read with great interest, does nothing to allay the suspicion. Mr. D'Arcy Power's article on "The Surgery of Syphilis," amid all its graces of diction and despite the curious interest of its historical research, has in many parts hints of "journalese" and an ephemeral character inconsistent with the style of a standard classic. Important as the frequent association and mutual interaction of tuberculosis and syphilis undoubtedly is, it seems to us that he drives his theme a little too hard. Almost exactly one-third of the article is devoted to treatment—a very just proportion if it were not followed by another eighty pages covering the same ground from the pen of another writer. What is the reason for this duplication? It looks very much as if, in his capacity as editor, Mr. D'Arcy Power had felt that in entrusting "The Treatment of Syphilis" to Colonel Lambkin he was inviting special pleading on behalf of metallic mercurial injections, and thought it wise, for the sake of the "System" idea, to be beforehand with an unbiassed account of other practice, which has the support of some of the greatest of syphilologists. With this, however, we have done grumbling, and hasten to recur to the interest with which everybody must read his account of syphilis of the joints, the breast, the prostate, the rectum, and other organs and tissues. Syphilis of the liver will probably be treated in another volume, but the diffuse form, seen in malarial subjects, is sometimes associated with such temperature and other symptoms as to lead experienced surgeons into a diagnosis of hepatic abscess and hence perhaps deserved mention.

Colonel Lambkin's article on treatment needs little comment; it embodies more fully what has been extensively published elsewhere, and makes out a very strong case; the abolition of pain after injections, even of calomel, which he claims to have effected by the use of absolute creosote and camphoric acid will certainly diminish the force of one argument against his views. His other contribution on "An Outbreak of Syphilis in a Virgin Soil" gives an account of the spread and checking of the disease in Uganda, and is pregnant with ethnographical and epidemiological interest. Dr. W. J. Gow contributes, in a short article of sixteen pages on "Syphilis in Obstetrics," a considered statement of prevalent views on conceptional syphilis and an account of the morbid anatomy of placental and fœtal tissues. It would appear that he is not quite in agreement with Colonel Lambkin as to the desirability of mercurialising a prospective mother, the wife of a syphilitic treated "for two years" immediately before his marriage. Some of the plates in this volume are very good, quite up to the standard set by those of vol. 1; but others can only be described as indifferent.

SYPHILIS IN THE ARMY. By Major H. C. FRENCH, R.A.M.C. (London : J. Bale, Sons and Danielsson, 1908. Royal 8vo. Pp. 126. 6s. net).

THAT Major French's book should consist of but three chapters and fifty-one appendices, mainly statistical, is a hint to the reader to read carefully. If all this pabulum has been thoroughly digested there must be a great deal for absorption. There is. Whilst rapid strides are being made towards the better understanding of the pathogeny of syphilis the time is ripening for a comprehensive scheme dealing with the scourge in the department of State medicine; and nowhere can the information that will be required be better obtained than from records, accurately kept on sound statistical methods, such as might be supplied by the medical department of an army having troops in all latitudes. The chapter on "Prevention" would be read with advantage by all who are engaged in the administration of the common weal. The problem to be grappled with in the face of the most various difficulties, with reference to the Army in India, is, if courage were found to face it, the problem of all great centres of population, and is perhaps more urgent than that of tuberculosis. Besides the discussion of administrative questions, however, the author has a chapter on treatment that deserves thoughtful consideration. He differs from some of the best-known authorities both as to the interpretation of clinical data and upon the relative value of certain methods of treatment. His opinions are obviously founded upon a solid basis of personal observations carefully recorded; as such they demand attention. We think that future writers upon the subject of syphilis will be indebted to this book; its perusal will stimulate many to reconsider certain features of their practice.

### THE DISEASES OF CHILDREN

Reports of the Society for the Study of Disease in Children. Edited by George Carpenter, M.D., with the assistance of a publication committee. Vol. VIII., Session 1907-8. (London : J. and A. Churchill. Pp. 504. Plates viii, illustrations in text 66. Price 12s. 6d. net.)

This handsome volume, brought out under the admirable editorship of Dr. G. Carpenter, more than maintains the reputation of the Society for the Study of Disease in Children for the production and publication of good work. Like the previous volumes, it is set up in excellent type, and is well and profusely illustrated, some of the plates being coloured. It is a larger volume than usual, for it is the last to be published by the Society as a body independent of the Royal Society of Medicine. As a fitting termination to his labours in the interests of the Society the editor has included a history of this body from its foundation in 1900 up to the date of amalgamation. The series of yearly reports should be in the hands of everyone interested in the affections of early life. They contain a most valuable record of almost all the recent advances in this special branch of medicine, discussions of very great interest and value, and reports of a large number of striking or characteristic cases; for the work of the Society has been largely directed to the clinical side of the subject rather than to abstruse points of pathology and bacteriology, though these have at times received sufficient consideration. The present volume is similar in character to previous ones, and it is impossible to draw attention to more than a few of the contributions. The most notable and the most useful ones are the Discussion on Inherited Syphilis, in which many members took part; papers on the Treatment of Congenital Hypertrophy of the Pylorus, by Drs. G. Carpenter and Edmund Cautley, and the discussion thereon; the Wightman Lecture, given by Sir Watson Cheyne on "The Defensive Arrangements of the Body as Illustrated by the Incidence of the Disease in Children and Adults"; the Prognosis of Heart Disease in Children, by Dr. Douglas Stanley; and on the Diagnosis of Urinary Tuberculosis in Children, with beautiful coloured plates, by Mr. C. A. Leedham-Green. We sincerely trust that the new section of the Royal Society of Medicine will be able to continue the annual publications of its proceedings in a like excellent form, for there is no doubt that this Society has very greatly stimulated the study and advanced the knowledge of this important subject.

Some Characteristics and Requirements of Childhood. By Alice Ravenhill, F.R.San.I. (Leeds and Glasgow : E. J. Arnold and Son. Pp. 70. Price 9d. net.)

Miss Ravenhill has collected into book form a series of articles which appeared in the *Guardian* during 1905-6, revised and partly rewritten. They include chapters on general characteristics of child life, requirements of infants' food, nurture, growth, and development, educational needs and play in childhood, and some characteristics of adolescence. Naturally these large subjects have been somewhat sketchily discussed in such a limited compass. The style is that of a leading article in a popular paper, and the matter stimulates interest in the subject more than it provides information. The writer has a remarkable taste for grandiloquent expression and sentences of inordinate length. A statement to which we take exception is that cow's milk contains only 2.5 per cent. of proteid. It is a work which every parent can read with advantage.

### ANÆSTHETICS.

Practical Points in Anæsthesia. By. F. Neef, M.D. (New York : Surgery Publishing Co., 1908. Price 60 cents).

Not so very long ago American medical opinion was much exercised over the backward state of the teaching and practice of anæsthetics in that continent; and to judge by some of the medical literature which reached this country there was some ground for mild pessimism. Lately, however, English anæsthetists have discovered that progress in their specialty is by no means a monopoly of the Old World, and have not been unwilling to learn a lesson from American anæsthetists in such matters as the "open ether" method. Speaking generally, chloroform and mixtures containing it have been much more employed in England than across the Atlantic, whereas ether as the ideal anæsthetic has had a greater vogue there than here. It is a little surprising, therefore, to find a New York anæsthetist whose invariable routine is to induce and maintain anæsthesia by chloroform or by anæsthol (a mixture of chloroform, ether, and ethyl chloride in molecular proportions), following a preliminary injection of a quarter of a grain of morphia. The points dealt with in the contents table offer a selection of problems not often fully dealt with in text-books on the subject, but of considerable practical interest. Unfortunately the handling of them is distinctly incomplete and superficial. There are, moreover, statements which are, in our judgment, positively incorrect. We do not feel that this book will be of value either to practitioners or specialists, nor that it will enhance in Great Britain the reputation of American work on this subject.

### OTOLOGY.

Infected Ears (Intrameatal Treatment). By F. Faulder White, F.R.C.S. (London : Celtic Press. 1908. Octavo. Pp. 100. Price 5s.)

The practitioner who takes up this monograph in the hope of finding guidance in the treatment of his cases of chronic aural suppuration will be sadly disappointed. Mr. Faulder White is well known as an enthusiastic advocate of intrameatal treatment, but though a great part of the little book is filled with the histories of his cases, one looks in vain for a coherent description of his technique. He has allowed his enthusiasm for one particular line of treatment to warp his sense of proportion, and he apparently believes that other otologists are ignorant of the value of antiseptic irrigation, and of intrameatal removal of granulations and carious bone. He disapproves of the radical mastoid operation in the treatment of chronic suppuration, and goes so far as to recommend "otectomy" in the treatment of otitic meningitis. The book is loosely written, and a great part consists of reports of cases.

### AMBULANCE WORK.

Aids to Memory for First-Aid Students. By L. M. F. Christian. (St. John Ambulance Association, 6d. net.)

This has already reached a second edition within six months of its first publication, and evidently fills a want. The work is prepared, the author says in the preface, to place before ambulance students at a glance all the principles of treatment as laid down in the official handbook of the St. John Ambulance Association. In other words it is comparable with the "Pocket Gray" of the anatomy student, and we should think it probably possesses both the merits and the defects of that popular synopsis. That is to say it is probably most useful as a last-minute examination book of revision, but not much calculated to lay the foundations of a lasting and practical knowledge of the subject. To criticise any of the details of this tabloid form of first-aid is, we may suppose, much the same as criticising the principles of treatment officially adopted by the St. John Association; but we cannot refrain from feeling some disagreement on certain of the points dealt with, nor from expressing it on the question of internal hæmorrhage. It is advised for this condition to "encourage better circulation in the brain" by various means, among which are mentioned smelling-salts, the sprinkling of water on the face, and the raising and bandaging of the limbs. We think the first two are pure waste of time and useless, and the last positively and directly harmful, as well as contrary to the most elementary principles of treatment. Further, in order to "excite contraction of the blood-vessels at the injured part," the patient is to be given ice to suck, cold water in sips, and an ice-bag or cold application over the suspected part; the combination of these remedies—no hint is given that they should not all be applied in every case—might, so it seems to us, be worse than the disease, or at least might make the disease worse. The headline of this section, in capitals, is "Assist Nature," with a reference to another page on which we can find nothing particularly germane. Mr. Christian's book suffers from over-condensation; so long as it is used simply as an aid to memory before an examination it is fulfilling a useful purpose; but as a substitute for a larger handbook in actual emergency we think its use would be unfortunate.

Supplementary First-Aid to Miners. By W. B. Arthur. (Bristol: J. Wright and Co., 6d.)

This is a small pamphlet designed as an adjunct to ordinary first-aid text-books and courses. It deals exclusively with the conditions and treatment of various accidents to which miners are especially liable, and indicates merely the general lines to be followed, not the actual details of first-aid technique. This plan is undoubtedly a wise one, as it avoids all chance of tempting mine-workers to neglect the fuller text-books on the subject. The instructions given are sound and practical, and the publication as a whole is a thoroughly useful one.

### ADVANCED PHYSIOLOGY.

Intracellular Enzymes. By H. M. Vernon, M.A., M.D. 8vo. Pp. xii.+240. (London: John Murray. Price 7s. 6d. net.)

This book embodies the subject-matter of a course of advanced lectures given in the Physiological Laboratory of the University of London, and constitutes another of the neatly bound and nicely printed blue-books that are being issued under the authority of that University. Much of the work is based upon researches made by Dr. Vernon himself. In addition to this the gist of the literature dealing with intracellular enzymes, scattered and fragmentary as it has hitherto been, is here collected together in a very convenient form. It has become increasingly clear of late years that there are a very large number of the vital phenomena of cell-nutrition and cell-growth that are explainable upon the hypothesis that various different ferments are at work. It is particularly during the last ten years that the ferments that are present within cells—as distinct from ferments such as pepsin, trypsin, ptyalin, amylopsin, and so forth, that have long been recognised in extracellular fluids—have been studied with scientific exactness; and if the rate of progress attained so far is continued in the future, the subject of intracellular enzymes bids fair to become, if it has not already become, one of the most important branches of bio-chemistry, for it alone seems to offer a clue to the solution of the most fundamental of all biological problems—the nature and constitution of living protoplasm. The author gives a clear account of the methods of investigating the various ferments, and of the evidence for and against the existence of, and the actions of, each. The power of reversible action possessed by enzymes is dealt with at some length, and many examples of synthesis in animals and plants are discussed. The *rôles* played by these various endo-enzymes in metabolism, and the lines of thought that recent discoveries have led to, are indicated as far as is at present possible. The action of intracellular enzymes in healthy cells would seem to be quite comparable to the action of the intracellular toxins of bacteria in causing disease. A full knowledge of the facts contained in Dr. Vernon's book will probably prove very helpful in elucidating the theories of bacteriologists, whilst in much the same way the investigations that bacteriologists are making in connection with immunity and the modes of action of bacteria must be helpful in elucidating the theories of ferment actions in healthy cells.

### DENTAL SURGERY.

Our Teeth: How Built Up; How Preserved; How Destroyed. By K. Denison Pedley, F.R.C.S. Ed., L.D.S. Eng., and Frank Harrison, M.R.C.S. Eng., L.D.S. Ed. Price 5s. net. (Blackie and Son, Limited.)

This book is written for the public with a view to conveying such information to parents and others that they may understand the nature of a tooth, why it decays, and what may be done in the way of prevention. The authors give a brief account of the embryological formation of the teeth, their development and eruption. They show what a normal dentition should be, and how bacteria bring about dental disease. Although we recognise the endeavour to keep the language simple, we fear the work will be found somewhat beyond the grasp of the lay mind, touching as it does on such subjects as embryology, histology, physiology, bacteriology, and pathology. Toxins and antitoxins, opsonins, and phagocytosis, pyæmia, and septicæmia all find a place here. In the hands of teachers with a knowledge of physiology, lecturers on hygiene, public health, and the upbringing of children, this book should serve admirably as the basis of lectures on oral hygiene and the care of the teeth. The illustrations are excellent. We should like to see the value of conservative dentistry, and the necessity of constant inspection brought out even more strongly, and we must dissent from the last statement in the book, "Clean teeth seldom decay."

### MISCELLANEOUS ITEMS.

Human Speech. By N. C. Macnamara, F.R.C.S. (Internat. Scientific Series. Vol. xcv. London: Kegan, Paul, Trench, Trübner and Co., 5s. net. 260 pages with numerous illustrations.)

This volume is in no way below the standard which the series demands. Although we find nothing in the book itself to call for unfavourable criticism, we cannot help thinking that the title is somewhat misleading. Mr. Macnamara, doubtless with the best intention in the world, conceives it his duty to trace zoologically the origin and development of the nervous system, as well as showing the lines of evolution from bacteria to metazoa. In fact only three chapters, properly speaking, are devoted to the subject which gives the book its title. The work throughout shows evidence of careful selection, in so far as the opinions of eminent scientists are frequently quoted. The illustrations, as the author says, are taken from standard text-books, and are therefore as accurate as can be obtained at present, without the trouble of doing original drawing. We are inclined to think that far too much prominence is given in chapter xii. to the deductions of Dr. Spitzka from an examination of the brains of six eminent Americans. The tendency of the general public is to take such conclusions and accept them as gospel, and thus increase the popular mass of fallacy and error. On the whole, the book is distinctly valuable for its suggestive capacity rather than for its demonstration of the function of speech organs. Its chief merit is its clearness of arrangement. The style, if unadorned, is restrained, and well suited for popular exposition. On the transmission of acquired characters, Mr. Macnamara observes: "To say that it (*i.e.* readjustment of structure to new incident force) is not inheritable is to say indirectly that force does not persist." We commend this to the attention of Mendelians.

The History of the Wine Trade in England. By André L. Simon, F.R. Hist. S. Two vols. (London: Wyman and Sons, Limited, 1906-1907.)

The first volume of M. André Simon's work on the history of the wine trade was issued more than a year ago; the second, and concluding volume, has just been published, and the two volumes may therefore be considered together. The amount of work, research, and patience that the author has expended on the production of the work must impress every reader on first taking up the volumes. A mass of documentary evidence has been digested, sifted, and carefully weighed for purposes of study and comparison; the result is an epitome of the history of the trade, which will remain for a long while the standard work on the subject, so far at least as the pre-Tudor period is concerned. We cannot here allow ourselves the pleasure of considering the work from a purely historical, archæological, and, what is even more interesting, from an etymological point of view, yet we cannot refrain from expressing our gratitude to the author for the light that he has thrown on many interesting and obscure points concerning the derivation of some of the words with which students of early English literature are acquainted. That light is always illuminating, and the arguments advanced by the author are usually impressive, even when not absolutely convincing. Thus he shows that the wines of St. John came, not from Johannisberg but from St. Jean d'Angely, and that the Rhenish wines of which Madox speaks were Moselle produce. Less strong is the case that he makes out on behalf of the plea that Osey wine was a Spanish vintage. Most commentators seem to think that this was a sour wine, the name being a corruption of *oseille*. We regret that space forbids us commenting on the many other interesting points raised in the book. The volumes are well worth reading, and medical men who desire to get an insight into the history of the wine trade in this country cannot do better than glance through some of the chapters, all of them interestingly written, in a discursive, chatty style, which makes some parts of them fascinating. The author tells much of the estimation in which wines were held, and gives some quaint medicinal prescriptions of which the various vintages formed the basis. The book is a valuable one, which should find a place in every library among reference works.

Glanders. By Wm. Hunting, F.R.C.V.S. (London: H. and W. Brown. Price 10s. 6d. net.)

We are not sure that we could mention off-hand a single infective disorder prevalent among animals to which has been devoted so elegant a monograph as this. Mr. Hunting is Chief Veterinary Inspector to the London County Council, in which position his opportunities of seeing this disease clinically must be quite unique, for, as is shown by tables of statistics, more than 90 per cent. of the cases of glanders in the kingdom occur in London and the home counties, chiefly in the former. His treatise is complete without diffuseness, scientific without being unpractical, and throughout eminently lucid. Of his appendix on glanders in man we will only say that we could wish it reproduced in text-books of medicine just as it stands. The book is excellently produced and illustrated with a series of coloured plates by Mr. S. A. Sewell which are well worthy of the letterpress. The full text is given of the 1907 Glanders and Farcy Order of the Board of Agriculture, which Mr. Hunting believes will, if properly enforced, entirely stamp out the disease in time. It is worthy of note that the Treasury refused the necessary financial assistance, and the expense has accordingly been put upon the local authorities.

